# Supplementary material for: Bioinformatics of cyanophycin metabolism genes and characterization of promiscuous isoaspartyl dipeptidases that catalyze the final step of cyanophycin degradation
Source: Sci Rep. 2023 May 23;13:8314. doi: 10.1038/s41598-023-34587-w (PMC10206079; doi:10.1038/s41598-023-34587-w)
Supplement: Supplementary file 1 — Supplementary Information 1. [file 41598_2023_34587_MOESM1_ESM.pdf]

Full wwPDB X-ray Structure Validation Report (i)

Jul 20, 2022 – 09:58 AM EDT

PDB ID	:	8DQN
Title	:	Crystal structure of isoaspartyl dipeptidase from Leucothrix mucor DSM2157
Deposited on	:	2022-07-19
Resolution	:	1.80 Å(reported)

This wwPDB validation report is for manuscript review

This is a Full wwPDB X-ray Structure Validation Report.

This report is produced by the wwPDB biocuration pipeline after annotation of the structure.

We welcome your comments at *validation@mail.wwpdb.org*
A user guide is available at
https://www.wwpdb.org/validation/2017/XrayValidationReportHelp
with specific help available everywhere you see the (i) symbol.

The types of validation reports are described at http://www.wwpdb.org/validation/2017/FAQs#types.

For Manuscript Review

The following versions of software and data (see references (i)) were used in the production of this report:

MolProbity	:	4.02b-467
Mogul	:	1.8.5 (274361), CSD as541be (2020)
Xtriage (Phenix)	:	1.13
EDS	:	2.29
Percentile statistics	:	20191225.v01 (using entries in the PDB archive December 25th 2019)
Refmac	:	5.8.0158
CCP4	:	7.0.044 (Gargrove)
Ideal geometry (proteins)	:	Engh & Huber (2001)
Ideal geometry (DNA, RNA)	:	Parkinson et al. (1996)
Validation Pipeline (wwPDB-VP)	:	2.29

1 Overall quality at a glance (i)

The following experimental techniques were used to determine the structure:
X-RAY DIFFRACTION

The reported resolution of this entry is 1.80 Å.

Percentile scores (ranging between 0-100) for global validation metrics of the entry are shown in the following graphic. The table shows the number of entries on which the scores are based.

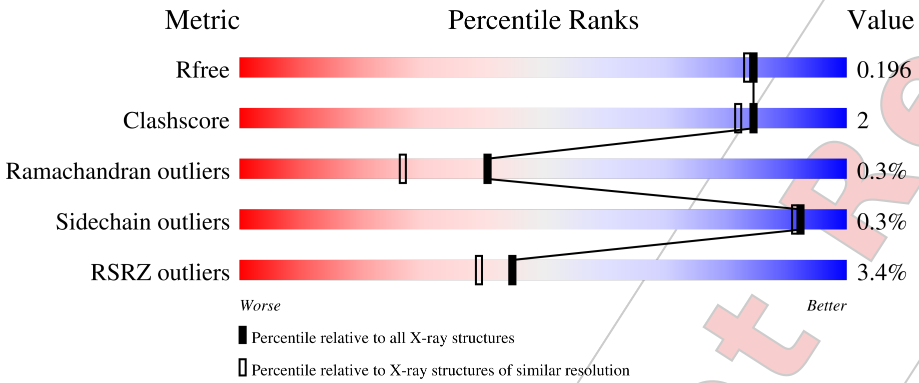

Metric	Whole archive (#Entries)	Similar resolution (#Entries, resolution range(Å))
R_{free}	130704	5950 (1.80-1.80)
Clashscore	141614	6793 (1.80-1.80)
Ramachandran outliers	138981	6697 (1.80-1.80)
Sidechain outliers	138945	6696 (1.80-1.80)
RSRZ outliers	127900	5850 (1.80-1.80)

The table below summarises the geometric issues observed across the polymeric chains and their fit to the electron density. The red, orange, yellow and green segments of the lower bar indicate the fraction of residues that contain outliers for >=3, 2, 1 and 0 types of geometric quality criteria respectively. A grey segment represents the fraction of residues that are not modelled. The numeric value for each fraction is indicated below the corresponding segment, with a dot representing fractions <=5% The upper red bar (where present) indicates the fraction of residues that have poor fit to the electron density. The numeric value is given above the bar.

Mol	Chain	Length	Quality of chain
1	A	394	2% / 91% 6% •
1	B	394	3% / 91% 5% •
1	C	394	4% / 93% 5% •
1	D	394	4% / 91% 5% •
1	E	394	4% / 92% • • •

Continued on next page...

For Manuscript Review

Continued from previous page...

Mol	Chain	Length	Quality of chain
1	F	394	4% 93% • •
1	G	394	3% 92% • •
1	H	394	3% 93% • •

The following table lists non-polymeric compounds, carbohydrate monomers and non-standard residues in protein, DNA, RNA chains that are outliers for geometric or electron-density-fit criteria:

Mol	Type	Chain	Res	Chirality	Geometry	Clashes	Electron density
3	ZN	B	403	-	-	-	X
3	ZN	H	403	-	-	-	X

For Manuscript Review

2 Entry composition (i)

There are 5 unique types of molecules in this entry. The entry contains 25943 atoms, of which 0 are hydrogens and 0 are deuteriums.

In the tables below, the ZeroOcc column contains the number of atoms modelled with zero occupancy, the AltConf column contains the number of residues with at least one atom in alternate conformation and the Trace column contains the number of residues modelled with at most 2 atoms.

- Molecule 1 is a protein called Isoaspartyl dipeptidase.

Mol	Chain	Residues	Atoms					ZeroOcc	AltConf	Trace
			Total	C	N	O	S			
1	A	383	2856	1809	487	546	14	0	0	0
1	B	380	2835	1794	484	543	14	0	0	0
1	C	388	2888	1826	492	556	14	0	0	0
1	D	380	2837	1796	484	543	14	0	0	0
1	E	381	2846	1801	485	546	14	0	0	0
1	F	384	2865	1814	488	549	14	0	0	0
1	G	379	2826	1787	483	542	14	0	0	0
1	H	380	2833	1792	484	543	14	0	0	0

- Molecule 2 is GLYCEROL (three-letter code: GOL) (formula: $C_3H_8O_3$).

For Manuscript Review

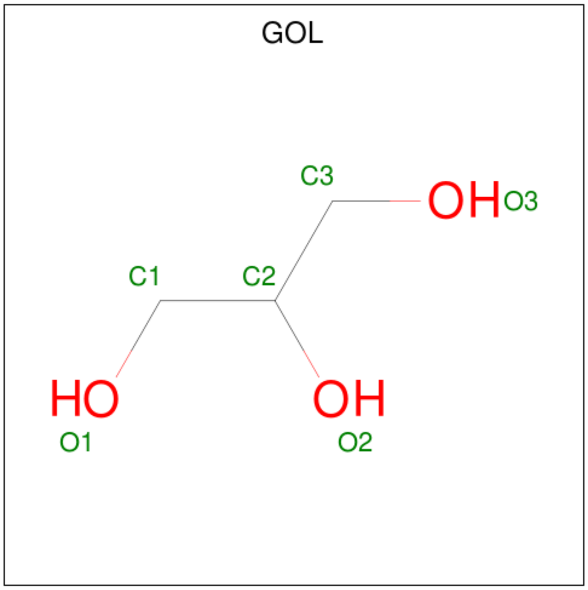

Mol	Chain	Residues	Atoms	ZeroOcc	AltConf
2	A	1	Total C O<br>6 3 3	0	0
2	B	1	Total C O<br>6 3 3	0	0
2	C	1	Total C O<br>6 3 3	0	0
2	D	1	Total C O<br>6 3 3	0	0
2	E	1	Total C O<br>6 3 3	0	0
2	F	1	Total C O<br>6 3 3	0	0
2	G	1	Total C O<br>6 3 3	0	0
2	H	1	Total C O<br>6 3 3	0	0

- Molecule 3 is ZINC ION (three-letter code: ZN) (formula: Zn).

Mol	Chain	Residues	Atoms	ZeroOcc	AltConf
3	A	2	Total Zn<br>2 2	0	0
3	B	2	Total Zn<br>2 2	0	0
3	C	2	Total Zn<br>2 2	0	0
3	D	2	Total Zn<br>2 2	0	0

Continued on next page...

For Manuscript Review

Continued from previous page...

Mol	Chain	Residues	Atoms	ZeroOcc	AltConf
3	E	2	Total Zn<br>2 2	0	0
3	F	2	Total Zn<br>2 2	0	0
3	G	2	Total Zn<br>2 2	0	0
3	H	2	Total Zn<br>2 2	0	0

- Molecule 4 is PHOSPHATE ION (three-letter code: PO4) (formula: O_4P).

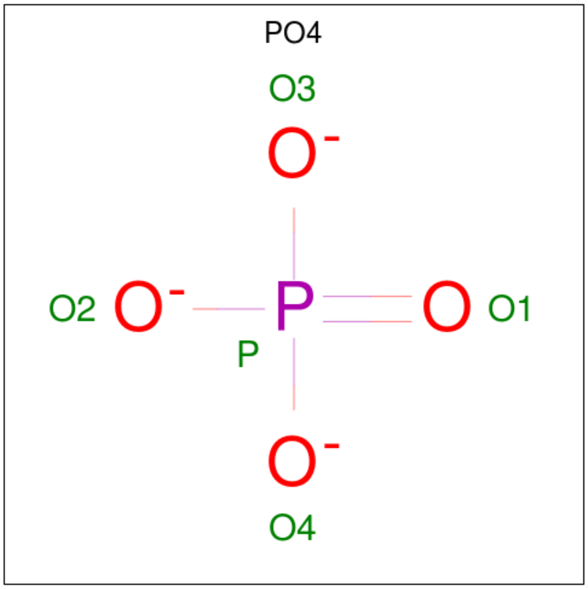

Mol	Chain	Residues	Atoms	ZeroOcc	AltConf
4	A	1	Total O P<br>5 4 1	0	0
4	B	1	Total O P<br>5 4 1	0	0
4	C	1	Total O P<br>5 4 1	0	0
4	D	1	Total O P<br>5 4 1	0	0
4	E	1	Total O P<br>5 4 1	0	0
4	F	1	Total O P<br>5 4 1	0	0
4	G	1	Total O P<br>5 4 1	0	0

Continued on next page...

For Manuscript Review

Continued from previous page...

Mol	Chain	Residues	Atoms	ZeroOcc	AltConf
4	H	1	Total O P<br>5 4 1	0	0

- Molecule 5 is water.

Mol	Chain	Residues	Atoms	ZeroOcc	AltConf
5	A	385	Total O<br>385 385	0	0
5	B	389	Total O<br>389 389	0	0
5	C	367	Total O<br>367 367	0	0
5	D	400	Total O<br>400 400	0	0
5	E	353	Total O<br>353 353	0	0
5	F	371	Total O<br>371 371	0	0
5	G	393	Total O<br>393 393	0	0
5	H	395	Total O<br>395 395	0	0

For Manuscript Review

3 Residue-property plots ⓘ

These plots are drawn for all protein, RNA, DNA and oligosaccharide chains in the entry. The first graphic for a chain summarises the proportions of the various outlier classes displayed in the second graphic. The second graphic shows the sequence view annotated by issues in geometry and electron density. Residues are color-coded according to the number of geometric quality criteria for which they contain at least one outlier: green = 0, yellow = 1, orange = 2 and red = 3 or more. A red dot above a residue indicates a poor fit to the electron density (RSRZ > 2). Stretches of 2 or more consecutive residues without any outlier are shown as a green connector. Residues present in the sample, but not in the model, are shown in grey.

- Molecule 1: Isoaspartyl dipeptidase

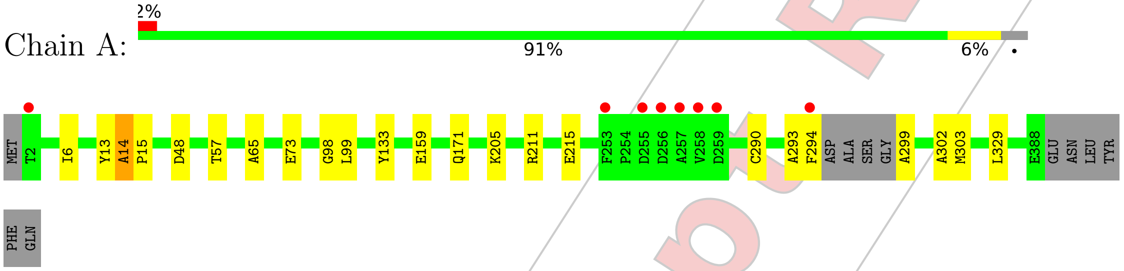

- Molecule 1: Isoaspartyl dipeptidase

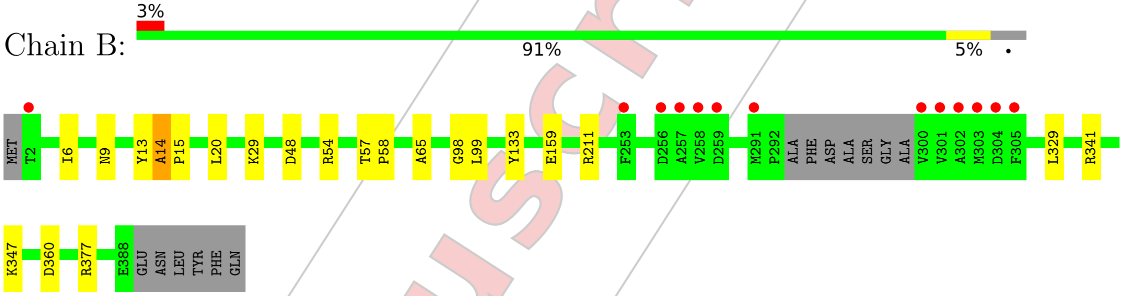

- Molecule 1: Isoaspartyl dipeptidase

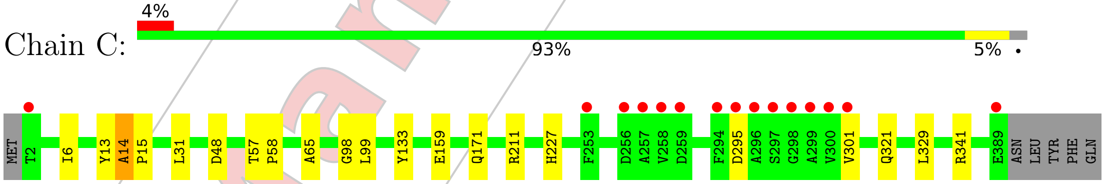

- Molecule 1: Isoaspartyl dipeptidase

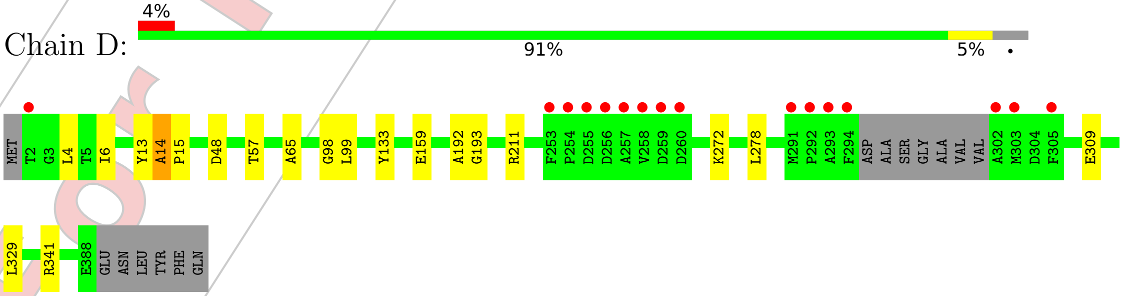

For Manuscript Review

• Molecule 1: Isoaspartyl dipeptidase

Chain E:

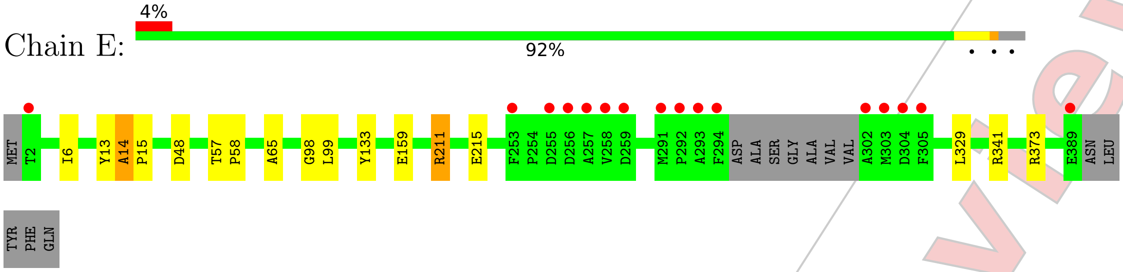

• Molecule 1: Isoaspartyl dipeptidase

Chain F:

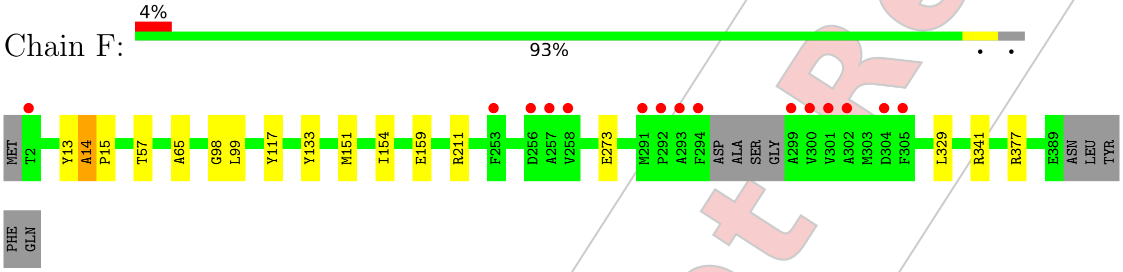

• Molecule 1: Isoaspartyl dipeptidase

Chain G:

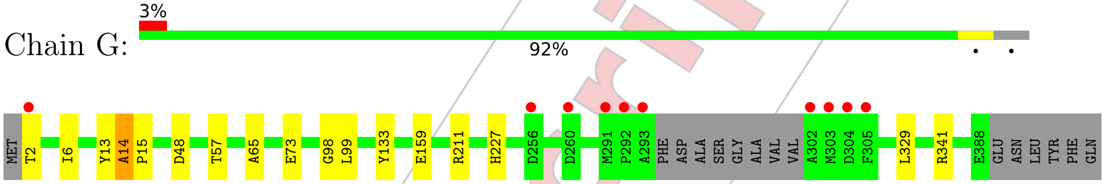

• Molecule 1: Isoaspartyl dipeptidase

Chain H:

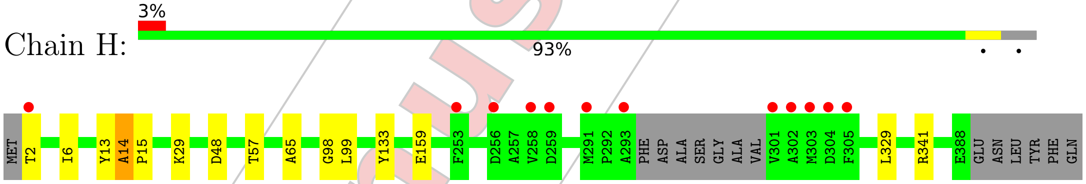

For Manuscript Review

4 Data and refinement statistics (i)

Property	Value	Source
Space group	P 21 21 21	Depositor
Cell constants<br>a, b, c, α, β, γ	153.49Å 163.69Å 170.45Å<br>90.00° 90.00° 90.00°	Depositor
Resolution (Å)	118.34 – 1.80<br>118.07 – 1.80	Depositor<br>EDS
% Data completeness<br>(in resolution range)	98.2 (118.34-1.80)<br>98.0 (118.07-1.80)	Depositor<br>EDS
R_{merge}	0.10	Depositor
R_{sym}	(Not available)	Depositor
$< I/\sigma(I) >$ [1]	1.02 (at 1.80Å)	Xtriage
Refinement program	REFMAC 5.8.0267	Depositor
R, R_{free}	0.166 , 0.187<br>0.176 , 0.196	Depositor<br>DCC
R_{free} test set	18657 reflections (4.83%)	wwPDB-VP
Wilson B-factor (Å^2)	26.4	Xtriage
Anisotropy	0.209	Xtriage
Bulk solvent k_{sol}(e/Å^3), B_{sol}(Å^2)	0.36 , 45.0	EDS
L-test for twinning[2]	$< \|L\| > = 0.49$, $< L^2 > = 0.31$	Xtriage
Estimated twinning fraction	0.016 for -h,l,k	Xtriage
F_o,F_c correlation	0.97	EDS
Total number of atoms	25943	wwPDB-VP
Average B, all atoms (Å^2)	32.0	wwPDB-VP

Xtriage's analysis on translational NCS is as follows: *The largest off-origin peak in the Patterson function is 3.15% of the height of the origin peak. No significant pseudotranslation is detected.*

[1] Intensities estimated from amplitudes.
[2] Theoretical values of $< |L| >$, $< L^2 >$ for acentric reflections are 0.5, 0.333 respectively for untwinned datasets, and 0.375, 0.2 for perfectly twinned datasets.

For Manuscript Review

5 Model quality (i)

5.1 Standard geometry (i)

Bond lengths and bond angles in the following residue types are not validated in this section: GOL, PO4, ZN

The Z score for a bond length (or angle) is the number of standard deviations the observed value is removed from the expected value. A bond length (or angle) with $|Z| > 5$ is considered an outlier worth inspection. RMSZ is the root-mean-square of all Z scores of the bond lengths (or angles).

Mol	Chain	Bond lengths		Bond angles	
		RMSZ	#|Z| >5	RMSZ	#|Z| >5
1	A	0.69	0/2908	0.83	2/3951 (0.1%)
1	B	0.69	0/2886	0.83	4/3921 (0.1%)
1	C	0.72	0/2941	0.82	4/3997 (0.1%)
1	D	0.71	1/2889 (0.0%)	0.88	6/3924 (0.2%)
1	E	0.69	0/2898	0.83	3/3936 (0.1%)
1	F	0.70	0/2917	0.84	4/3963 (0.1%)
1	G	0.69	0/2877	0.83	3/3908 (0.1%)
1	H	0.70	0/2884	0.82	1/3918 (0.0%)
All	All	0.70	1/23200 (0.0%)	0.84	27/31518 (0.1%)

All (1) bond length outliers are listed below:

Mol	Chain	Res	Type	Atoms	Z	Observed(Å)	Ideal(Å)
1	D	193	GLY	N-CA	6.17	1.55	1.46

All (27) bond angle outliers are listed below:

Mol	Chain	Res	Type	Atoms	Z	Observed(^{o})	Ideal(^{o})
1	D	192	ALA	CA-C-N	10.14	136.48	116.20
1	D	192	ALA	C-N-CA	-9.44	102.47	122.30
1	D	192	ALA	O-C-N	-9.20	107.56	123.20
1	E	211	ARG	CG-CD-NE	-7.55	95.94	111.80
1	F	211	ARG	CG-CD-NE	-7.35	96.37	111.80
1	F	211	ARG	NE-CZ-NH2	-7.21	116.69	120.30
1	D	211	ARG	CG-CD-NE	-7.16	96.77	111.80
1	C	211	ARG	CG-CD-NE	-7.10	96.90	111.80
1	B	211	ARG	CG-CD-NE	-7.04	97.01	111.80
1	B	377	ARG	NE-CZ-NH2	-6.99	116.81	120.30
1	A	211	ARG	CG-CD-NE	-6.78	97.56	111.80
1	B	211	ARG	NE-CZ-NH2	-6.75	116.93	120.30

Continued on next page...

For Manuscript Review

Continued from previous page...

Mol	Chain	Res	Type	Atoms	Z	Observed(^{o})	Ideal(^{o})
1	G	211	ARG	CG-CD-NE	-6.70	97.73	111.80
1	E	341	ARG	NE-CZ-NH1	6.57	123.58	120.30
1	E	211	ARG	NE-CZ-NH2	-6.54	117.03	120.30
1	B	341	ARG	NE-CZ-NH2	-6.13	117.24	120.30
1	F	377	ARG	NE-CZ-NH2	-6.12	117.24	120.30
1	G	341	ARG	NE-CZ-NH1	6.09	123.34	120.30
1	F	341	ARG	NE-CZ-NH1	6.00	123.30	120.30
1	D	341	ARG	NE-CZ-NH1	5.80	123.20	120.30
1	D	193	GLY	CA-C-O	-5.63	110.47	120.60
1	C	211	ARG	NE-CZ-NH2	-5.61	117.50	120.30
1	A	211	ARG	NE-CZ-NH2	-5.42	117.59	120.30
1	C	341	ARG	NE-CZ-NH2	-5.31	117.64	120.30
1	C	341	ARG	NE-CZ-NH1	5.22	122.91	120.30
1	G	211	ARG	NE-CZ-NH2	-5.20	117.70	120.30
1	H	341	ARG	NE-CZ-NH1	5.17	122.89	120.30

There are no chirality outliers.

There are no planarity outliers.

5.2 Too-close contacts (i)

In the following table, the Non-H and H(model) columns list the number of non-hydrogen atoms and hydrogen atoms in the chain respectively. The H(added) column lists the number of hydrogen atoms added and optimized by MolProbity. The Clashes column lists the number of clashes within the asymmetric unit, whereas Symm-Clashes lists symmetry-related clashes.

Mol	Chain	Non-H	H(model)	H(added)	Clashes	Symm-Clashes
1	A	2856	0	2866	16	0
1	B	2835	0	2847	14	0
1	C	2888	0	2890	16	0
1	D	2837	0	2843	12	0
1	E	2846	0	2849	12	0
1	F	2865	0	2872	11	0
1	G	2826	0	2834	12	0
1	H	2833	0	2843	11	0
2	A	6	0	8	0	0
2	B	6	0	8	0	0
2	C	6	0	8	0	0
2	D	6	0	8	0	0
2	E	6	0	8	1	0
2	F	6	0	8	0	0

Continued on next page...

For Manuscript Review

Continued from previous page...

Mol	Chain	Non-H	H(model)	H(added)	Clashes	Symm-Clashes
2	G	6	0	8	0	0
2	H	6	0	8	0	0
3	A	2	0	0	0	0
3	B	2	0	0	0	0
3	C	2	0	0	0	0
3	D	2	0	0	0	0
3	E	2	0	0	0	0
3	F	2	0	0	0	0
3	G	2	0	0	0	0
3	H	2	0	0	0	0
4	A	5	0	0	0	0
4	B	5	0	0	0	0
4	C	5	0	0	0	0
4	D	5	0	0	0	0
4	E	5	0	0	0	0
4	F	5	0	0	0	0
4	G	5	0	0	0	0
4	H	5	0	0	0	0
5	A	385	0	0	6	0
5	B	389	0	0	4	0
5	C	367	0	0	6	0
5	D	400	0	0	3	1
5	E	353	0	0	3	1
5	F	371	0	0	3	0
5	G	393	0	0	5	1
5	H	395	0	0	4	1
All	All	25943	0	22908	103	2

The all-atom clashscore is defined as the number of clashes found per 1000 atoms (including hydrogen atoms). The all-atom clashscore for this structure is 2.

All (103) close contacts within the same asymmetric unit are listed below, sorted by their clash magnitude.

Atom-1	Atom-2	Interatomic distance (Å)	Clash overlap (Å)
1:C:133:TYR:HE2	5:C:572:HOH:O	1.00	1.31
1:G:133:TYR:HE2	5:G:616:HOH:O	1.17	1.26
1:A:133:TYR:HE2	5:A:559:HOH:O	0.97	1.25
1:H:133:TYR:HE2	5:H:587:HOH:O	1.12	1.23
1:E:133:TYR:HE2	5:E:603:HOH:O	1.19	1.23
1:B:133:TYR:CE2	5:B:510:HOH:O	2.05	1.10
1:B:133:TYR:HE2	5:B:510:HOH:O	1.32	1.08

Continued on next page...

For Manuscript Review

Continued from previous page...

Atom-1	Atom-2	Interatomic distance (Å)	Clash overlap (Å)
1:E:133:TYR:CE2	5:E:603:HOH:O	1.96	1.08
1:H:133:TYR:CE2	5:H:587:HOH:O	1.91	1.06
1:C:133:TYR:CE2	5:C:572:HOH:O	1.83	1.01
1:G:133:TYR:CE2	5:G:616:HOH:O	1.97	1.00
1:F:133:TYR:CE2	5:F:512:HOH:O	2.12	0.99
1:A:133:TYR:CE2	5:A:559:HOH:O	1.80	0.99
1:F:133:TYR:HE2	5:F:512:HOH:O	1.47	0.92
1:D:133:TYR:CE2	5:D:504:HOH:O	2.28	0.87
1:F:273:GLU:OE2	5:F:501:HOH:O	2.05	0.75
1:D:133:TYR:HE2	5:D:504:HOH:O	1.68	0.74
1:B:54:ARG:NH1	1:B:360:ASP:OD1	2.21	0.70
1:A:205:LYS:HE3	5:A:776:HOH:O	1.97	0.63
1:B:9:ASN:HA	1:B:20:LEU:HD11	1.81	0.62
1:A:73:GLU:HB3	5:A:503:HOH:O	2.07	0.54
1:C:171:GLN:HG3	5:C:836:HOH:O	2.07	0.54
1:E:373:ARG:HD3	5:E:798:HOH:O	2.10	0.52
1:A:293:ALA:HB3	1:A:302:ALA:HB3	1.92	0.51
1:A:13:TYR:C	1:A:15:PRO:HA	2.31	0.51
1:A:171:GLN:HG3	5:A:856:HOH:O	2.09	0.51
1:C:13:TYR:C	1:C:15:PRO:HA	2.31	0.51
1:E:159:GLU:O	1:E:159:GLU:HG3	2.11	0.51
1:G:13:TYR:C	1:G:15:PRO:HA	2.31	0.51
1:D:13:TYR:C	1:D:15:PRO:HA	2.31	0.51
1:D:159:GLU:HG3	1:D:159:GLU:O	2.11	0.51
1:B:347:LYS:HD3	5:B:663:HOH:O	2.10	0.51
1:B:13:TYR:C	1:B:15:PRO:HA	2.31	0.51
1:H:159:GLU:HG3	1:H:159:GLU:O	2.11	0.51
1:G:2:THR:HG22	5:G:841:HOH:O	2.11	0.50
1:G:159:GLU:O	1:G:159:GLU:HG3	2.11	0.50
1:F:13:TYR:C	1:F:15:PRO:HA	2.32	0.50
1:A:159:GLU:O	1:A:159:GLU:HG3	2.12	0.50
1:F:159:GLU:O	1:F:159:GLU:HG3	2.11	0.50
1:H:13:TYR:C	1:H:15:PRO:HA	2.32	0.50
1:C:159:GLU:O	1:C:159:GLU:HG3	2.12	0.50
1:E:13:TYR:C	1:E:15:PRO:HA	2.32	0.50
1:B:159:GLU:O	1:B:159:GLU:HG3	2.12	0.49
1:C:227:HIS:CG	5:C:582:HOH:O	2.65	0.49
1:C:295:ASP:HB3	1:C:301:VAL:HG21	1.93	0.49
2:E:401:GOL:H31	1:F:117:TYR:CD1	2.48	0.49
1:A:14:ALA:N	1:A:15:PRO:CA	2.77	0.48
1:C:14:ALA:N	1:C:15:PRO:CA	2.77	0.48

Continued on next page...

For Manuscript Review

Continued from previous page...

Atom-1	Atom-2	Interatomic distance (Å)	Clash overlap (Å)
1:D:309:GLU:HG3	5:D:812:HOH:O	2.14	0.47
1:C:14:ALA:N	1:C:15:PRO:HA	2.30	0.47
1:G:14:ALA:N	1:G:15:PRO:CA	2.77	0.47
1:G:227:HIS:CG	5:G:578:HOH:O	2.67	0.47
1:B:14:ALA:N	1:B:15:PRO:CA	2.78	0.47
1:A:14:ALA:N	1:A:15:PRO:HA	2.30	0.47
1:B:14:ALA:N	1:B:15:PRO:HA	2.30	0.47
1:F:14:ALA:N	1:F:15:PRO:CA	2.78	0.47
1:F:14:ALA:N	1:F:15:PRO:HA	2.30	0.46
1:G:14:ALA:N	1:G:15:PRO:HA	2.30	0.46
1:G:73:GLU:OE1	5:G:501:HOH:O	2.20	0.46
1:H:14:ALA:N	1:H:15:PRO:CA	2.79	0.46
1:E:14:ALA:N	1:E:15:PRO:CA	2.78	0.46
1:D:14:ALA:N	1:D:15:PRO:HA	2.31	0.46
1:D:14:ALA:N	1:D:15:PRO:CA	2.78	0.46
1:B:65:ALA:O	1:B:98:GLY:HA2	2.16	0.45
1:F:65:ALA:O	1:F:98:GLY:HA2	2.16	0.45
1:G:65:ALA:O	1:G:98:GLY:HA2	2.17	0.45
1:A:215:GLU:OE1	5:A:501:HOH:O	2.21	0.45
1:E:14:ALA:N	1:E:15:PRO:HA	2.31	0.45
1:H:14:ALA:N	1:H:15:PRO:HA	2.32	0.45
1:A:65:ALA:O	1:A:98:GLY:HA2	2.17	0.45
1:H:57:THR:HB	1:H:329:LEU:HD21	1.99	0.45
1:E:65:ALA:O	1:E:98:GLY:HA2	2.17	0.44
1:C:65:ALA:O	1:C:98:GLY:HA2	2.18	0.44
1:G:57:THR:HB	1:G:329:LEU:HD21	1.99	0.44
1:A:6:ILE:HG23	1:A:48:ASP:HA	2.00	0.44
1:H:65:ALA:O	1:H:98:GLY:HA2	2.17	0.44
1:C:57:THR:HB	1:C:329:LEU:HD21	2.00	0.43
1:B:57:THR:HB	1:B:329:LEU:HD21	2.01	0.43
1:D:6:ILE:HG23	1:D:48:ASP:HA	2.00	0.43
1:H:2:THR:HG22	5:H:723:HOH:O	2.17	0.43
1:C:321:GLN:HG3	5:C:752:HOH:O	2.18	0.43
1:B:29:LYS:HE3	5:B:624:HOH:O	2.19	0.43
1:E:57:THR:HB	1:E:329:LEU:HD21	2.00	0.43
1:F:57:THR:HB	1:F:329:LEU:HD21	2.01	0.43
1:D:65:ALA:O	1:D:98:GLY:HA2	2.18	0.43
1:B:6:ILE:HG23	1:B:48:ASP:HA	2.00	0.43
1:A:57:THR:HB	1:A:329:LEU:HD21	2.01	0.43
1:C:6:ILE:HG23	1:C:48:ASP:HA	2.01	0.43
1:E:6:ILE:HG23	1:E:48:ASP:HA	2.01	0.43

Continued on next page...

For Manuscript Review

Continued from previous page...

Atom-1	Atom-2	Interatomic distance (Å)	Clash overlap (Å)
1:D:57:THR:HB	1:D:329:LEU:HD21	2.01	0.42
1:E:13:TYR:O	1:E:58:PRO:HD3	2.20	0.42
1:C:31:LEU:HD13	1:D:4:LEU:HD21	2.02	0.42
1:G:6:ILE:HG23	1:G:48:ASP:HA	2.01	0.42
1:C:321:GLN:CG	5:C:752:HOH:O	2.66	0.42
1:H:6:ILE:HG23	1:H:48:ASP:HA	2.02	0.41
1:A:294:PHE:HA	1:A:299:ALA:O	2.20	0.41
1:E:211:ARG:HG3	1:E:215:GLU:OE2	2.21	0.41
1:C:13:TYR:O	1:C:58:PRO:HD3	2.21	0.40
1:F:151:MET:HG3	1:F:154:ILE:HD12	2.03	0.40
1:A:290:CYS:SG	1:A:303:MET:SD	3.19	0.40
1:B:13:TYR:O	1:B:58:PRO:HD3	2.22	0.40
1:H:29:LYS:HE2	5:H:653:HOH:O	2.21	0.40
1:D:272:LYS:HD3	1:D:278:LEU:HD11	2.03	0.40

All (2) symmetry-related close contacts are listed below. The label for Atom-2 includes the symmetry operator and encoded unit-cell translations to be applied.

Atom-1	Atom-2	Interatomic distance (Å)	Clash overlap (Å)
5:D:506:HOH:O	5:G:733:HOH:O[4_445]	2.11	0.09
5:E:709:HOH:O	5:H:693:HOH:O[4_545]	2.18	0.02

5.3 Torsion angles (i)

5.3.1 Protein backbone (i)

In the following table, the Percentiles column shows the percent Ramachandran outliers of the chain as a percentile score with respect to all X-ray entries followed by that with respect to entries of similar resolution.

The Analysed column shows the number of residues for which the backbone conformation was analysed, and the total number of residues.

Mol	Chain	Analysed	Favoured	Allowed	Outliers	Percentiles	
1	A	379/394 (96%)	368 (97%)	10 (3%)	1 (0%)	41	27
1	B	376/394 (95%)	367 (98%)	8 (2%)	1 (0%)	41	27
1	C	386/394 (98%)	375 (97%)	10 (3%)	1 (0%)	41	27
1	D	376/394 (95%)	364 (97%)	11 (3%)	1 (0%)	41	27

Continued on next page...

For Manuscript Review

Continued from previous page...

Mol	Chain	Analysed	Favoured	Allowed	Outliers	Percentiles	
1	E	377/394 (96%)	366 (97%)	10 (3%)	1 (0%)	41	27
1	F	380/394 (96%)	370 (97%)	9 (2%)	1 (0%)	41	27
1	G	375/394 (95%)	365 (97%)	9 (2%)	1 (0%)	41	27
1	H	376/394 (95%)	365 (97%)	10 (3%)	1 (0%)	41	27
All	All	3025/3152 (96%)	2940 (97%)	77 (2%)	8 (0%)	41	27

All (8) Ramachandran outliers are listed below:

Mol	Chain	Res	Type
1	B	14	ALA
1	A	14	ALA
1	C	14	ALA
1	D	14	ALA
1	E	14	ALA
1	F	14	ALA
1	G	14	ALA
1	H	14	ALA

5.3.2 Protein sidechains (i)

In the following table, the Percentiles column shows the percent sidechain outliers of the chain as a percentile score with respect to all X-ray entries followed by that with respect to entries of similar resolution.

The Analysed column shows the number of residues for which the sidechain conformation was analysed, and the total number of residues.

Mol	Chain	Analysed	Rotameric	Outliers	Percentiles	
1	A	299/308 (97%)	298 (100%)	1 (0%)	92	91
1	B	298/308 (97%)	297 (100%)	1 (0%)	92	91
1	C	302/308 (98%)	301 (100%)	1 (0%)	92	91
1	D	297/308 (96%)	296 (100%)	1 (0%)	92	91
1	E	298/308 (97%)	297 (100%)	1 (0%)	92	91
1	F	300/308 (97%)	299 (100%)	1 (0%)	92	91
1	G	296/308 (96%)	295 (100%)	1 (0%)	92	91
1	H	297/308 (96%)	296 (100%)	1 (0%)	92	91
All	All	2387/2464 (97%)	2379 (100%)	8 (0%)	92	91

For Manuscript Review

All (8) residues with a non-rotameric sidechain are listed below:

Mol	Chain	Res	Type
1	A	99	LEU
1	B	99	LEU
1	C	99	LEU
1	D	99	LEU
1	E	99	LEU
1	F	99	LEU
1	G	99	LEU
1	H	99	LEU

Sometimes sidechains can be flipped to improve hydrogen bonding and reduce clashes. All (9) such sidechains are listed below:

Mol	Chain	Res	Type
1	A	338	HIS
1	B	338	HIS
1	C	338	HIS
1	D	224	ASN
1	D	338	HIS
1	E	338	HIS
1	F	338	HIS
1	G	338	HIS
1	H	338	HIS

5.3.3 RNA (i)

There are no RNA molecules in this entry.

5.4 Non-standard residues in protein, DNA, RNA chains (i)

There are no non-standard protein/DNA/RNA residues in this entry.

5.5 Carbohydrates (i)

There are no monosaccharides in this entry.

5.6 Ligand geometry (i)

Of 32 ligands modelled in this entry, 16 are monoatomic - leaving 16 for Mogul analysis.

For Manuscript Review

In the following table, the Counts columns list the number of bonds (or angles) for which Mogul statistics could be retrieved, the number of bonds (or angles) that are observed in the model and the number of bonds (or angles) that are defined in the Chemical Component Dictionary. The Link column lists molecule types, if any, to which the group is linked. The Z score for a bond length (or angle) is the number of standard deviations the observed value is removed from the expected value. A bond length (or angle) with $|Z| > 2$ is considered an outlier worth inspection. RMSZ is the root-mean-square of all Z scores of the bond lengths (or angles).

Mol	Type	Chain	Res	Link	Bond lengths			Bond angles		
					Counts	RMSZ	$\#\|Z\| > 2$	Counts	RMSZ	$\#\|Z\| > 2$
4	PO4	B	404	-	4,4,4	0.85	0	6,6,6	0.55	0
2	GOL	B	401	-	5,5,5	0.23	0	5,5,5	0.61	0
4	PO4	C	404	-	4,4,4	0.89	0	6,6,6	0.49	0
2	GOL	A	401	-	5,5,5	0.06	0	5,5,5	0.35	0
4	PO4	E	404	-	4,4,4	0.21	0	6,6,6	0.66	0
4	PO4	H	404	-	4,4,4	0.83	0	6,6,6	0.40	0
2	GOL	H	401	-	5,5,5	0.17	0	5,5,5	0.47	0
4	PO4	G	404	-	4,4,4	1.09	0	6,6,6	0.44	0
2	GOL	C	401	-	5,5,5	0.17	0	5,5,5	0.80	0
4	PO4	A	404	-	4,4,4	1.18	1 (25%)	6,6,6	0.45	0
4	PO4	F	404	-	4,4,4	1.01	0	6,6,6	0.68	0
2	GOL	G	401	-	5,5,5	0.17	0	5,5,5	0.31	0
2	GOL	D	401	-	5,5,5	0.11	0	5,5,5	0.21	0
4	PO4	D	404	-	4,4,4	0.96	0	6,6,6	0.63	0
2	GOL	F	401	-	5,5,5	0.19	0	5,5,5	0.58	0
2	GOL	E	401	-	5,5,5	0.22	0	5,5,5	0.96	0

In the following table, the Chirals column lists the number of chiral outliers, the number of chiral centers analysed, the number of these observed in the model and the number defined in the Chemical Component Dictionary. Similar counts are reported in the Torsion and Rings columns. '-' means no outliers of that kind were identified.

Mol	Type	Chain	Res	Link	Chirals	Torsions	Rings
2	GOL	B	401	-	-	0/4/4/4	-
2	GOL	A	401	-	-	0/4/4/4	-
2	GOL	H	401	-	-	2/4/4/4	-
2	GOL	C	401	-	-	0/4/4/4	-
2	GOL	G	401	-	-	2/4/4/4	-
2	GOL	D	401	-	-	2/4/4/4	-
2	GOL	F	401	-	-	2/4/4/4	-
2	GOL	E	401	-	-	2/4/4/4	-

All (1) bond length outliers are listed below:

For Manuscript Review

Mol	Chain	Res	Type	Atoms	Z	Observed(Å)	Ideal(Å)
4	A	404	PO4	P-O1	2.06	1.55	1.50

There are no bond angle outliers.

There are no chirality outliers.

All (10) torsion outliers are listed below:

Mol	Chain	Res	Type	Atoms
2	D	401	GOL	O1-C1-C2-C3
2	E	401	GOL	C1-C2-C3-O3
2	F	401	GOL	O1-C1-C2-C3
2	G	401	GOL	O1-C1-C2-C3
2	H	401	GOL	O1-C1-C2-O2
2	H	401	GOL	O1-C1-C2-C3
2	D	401	GOL	O1-C1-C2-O2
2	E	401	GOL	O2-C2-C3-O3
2	G	401	GOL	O1-C1-C2-O2
2	F	401	GOL	O1-C1-C2-O2

There are no ring outliers.

1 monomer is involved in 1 short contact:

Mol	Chain	Res	Type	Clashes	Symm-Clashes
2	E	401	GOL	1	0

5.7 Other polymers (i)

There are no such residues in this entry.

5.8 Polymer linkage issues (i)

There are no chain breaks in this entry.

For Manuscript Review

6 Fit of model and data (i)

6.1 Protein, DNA and RNA chains (i)

In the following table, the column labelled '#RSRZ> 2' contains the number (and percentage) of RSRZ outliers, followed by percent RSRZ outliers for the chain as percentile scores relative to all X-ray entries and entries of similar resolution. The OWAB column contains the minimum, median, 95th percentile and maximum values of the occupancy-weighted average B-factor per residue. The column labelled 'Q< 0.9' lists the number of (and percentage) of residues with an average occupancy less than 0.9.

Mol	Chain	Analysed	<RSRZ>	#RSRZ>2			OWAB(Å^2)	Q<0.9
1	A	383/394 (97%)	-0.28	8 (2%)	63	59	19, 27, 50, 114	0
1	B	380/394 (96%)	-0.25	13 (3%)	45	39	19, 27, 58, 111	0
1	C	388/394 (98%)	-0.21	15 (3%)	39	33	18, 26, 67, 98	0
1	D	380/394 (96%)	-0.23	16 (4%)	36	30	18, 26, 55, 116	0
1	E	381/394 (96%)	-0.22	16 (4%)	36	30	18, 29, 59, 117	0
1	F	384/394 (97%)	-0.16	15 (3%)	39	33	19, 27, 64, 130	0
1	G	379/394 (96%)	-0.30	10 (2%)	56	51	18, 26, 52, 94	0
1	H	380/394 (96%)	-0.27	12 (3%)	47	41	18, 26, 55, 98	0
All	All	3055/3152 (96%)	-0.24	105 (3%)	45	39	18, 27, 59, 130	0

All (105) RSRZ outliers are listed below:

Mol	Chain	Res	Type	RSRZ
1	F	299	ALA	11.3
1	F	294	PHE	10.8
1	A	255	ASP	8.2
1	F	300	VAL	8.0
1	B	300	VAL	7.2
1	F	301	VAL	7.2
1	B	302	ALA	6.9
1	D	294	PHE	6.9
1	E	294	PHE	6.6
1	D	302	ALA	6.4
1	C	258	VAL	5.8
1	A	256	ASP	5.6
1	C	257	ALA	5.3
1	H	258	VAL	5.3
1	E	2	THR	5.1
1	C	298	GLY	5.1

Continued on next page...

For Manuscript Review

Continued from previous page...

Mol	Chain	Res	Type	RSRZ
1	A	2	THR	4.9
1	B	305	PHE	4.9
1	D	258	VAL	4.8
1	F	293	ALA	4.7
1	D	293	ALA	4.7
1	B	301	VAL	4.6
1	H	302	ALA	4.5
1	C	256	ASP	4.4
1	H	291	MET	4.4
1	C	301	VAL	4.4
1	F	302	ALA	4.3
1	B	2	THR	4.3
1	F	291	MET	4.3
1	A	257	ALA	4.2
1	B	291	MET	4.1
1	G	2	THR	4.0
1	C	294	PHE	3.9
1	D	253	PHE	3.8
1	C	253	PHE	3.8
1	F	2	THR	3.7
1	C	295	ASP	3.7
1	B	257	ALA	3.7
1	H	293	ALA	3.7
1	H	303	MET	3.6
1	C	259	ASP	3.6
1	F	258	VAL	3.6
1	H	301	VAL	3.5
1	F	305	PHE	3.5
1	C	2	THR	3.5
1	D	291	MET	3.4
1	E	258	VAL	3.3
1	C	389	GLU	3.3
1	D	257	ALA	3.2
1	D	260	ASP	3.2
1	B	304	ASP	3.2
1	B	258	VAL	3.2
1	G	291	MET	3.1
1	B	259	ASP	3.1
1	F	256	ASP	3.1
1	A	253	PHE	3.1
1	D	256	ASP	3.1
1	D	305	PHE	3.1

Continued on next page...

For Manuscript Review

Continued from previous page...

Mol	Chain	Res	Type	RSRZ
1	D	259	ASP	3.1
1	G	304	ASP	3.0
1	A	258	VAL	3.0
1	C	300	VAL	3.0
1	H	304	ASP	3.0
1	C	296	ALA	3.0
1	D	303	MET	3.0
1	F	292	PRO	2.9
1	E	302	ALA	2.9
1	G	256	ASP	2.9
1	E	256	ASP	2.9
1	E	253	PHE	2.9
1	H	305	PHE	2.8
1	G	305	PHE	2.8
1	E	389	GLU	2.7
1	D	255	ASP	2.7
1	F	257	ALA	2.7
1	C	297	SER	2.6
1	G	303	MET	2.6
1	H	2	THR	2.6
1	H	259	ASP	2.5
1	D	292	PRO	2.5
1	B	256	ASP	2.5
1	F	253	PHE	2.5
1	H	253	PHE	2.5
1	G	292	PRO	2.4
1	H	256	ASP	2.4
1	A	294	PHE	2.4
1	G	260	ASP	2.3
1	B	303	MET	2.3
1	B	253	PHE	2.3
1	E	303	MET	2.3
1	D	2	THR	2.3
1	E	257	ALA	2.3
1	G	302	ALA	2.3
1	E	304	ASP	2.3
1	E	255	ASP	2.2
1	C	299	ALA	2.2
1	E	291	MET	2.2
1	A	259	ASP	2.1
1	E	259	ASP	2.1
1	E	305	PHE	2.1

Continued on next page...

For Manuscript Review

Continued from previous page...

Mol	Chain	Res	Type	RSRZ
1	E	293	ALA	2.1
1	D	254	PRO	2.1
1	E	292	PRO	2.1
1	G	293	ALA	2.0
1	F	304	ASP	2.0

6.2 Non-standard residues in protein, DNA, RNA chains (i)

There are no non-standard protein/DNA/RNA residues in this entry.

6.3 Carbohydrates (i)

There are no monosaccharides in this entry.

6.4 Ligands (i)

In the following table, the Atoms column lists the number of modelled atoms in the group and the number defined in the chemical component dictionary. The B-factors column lists the minimum, median, 95th percentile and maximum values of B factors of atoms in the group. The column labelled 'Q< 0.9' lists the number of atoms with occupancy less than 0.9.

Mol	Type	Chain	Res	Atoms	RSCC	RSR	B-factors(Å^2)	Q<0.9
3	ZN	G	403	1/1	0.49	0.34	100,100,100,100	0
3	ZN	B	403	1/1	0.57	0.46	105,105,105,105	0
3	ZN	H	403	1/1	0.63	0.45	113,113,113,113	0
3	ZN	D	403	1/1	0.81	0.44	96,96,96,96	0
3	ZN	A	403	1/1	0.85	0.53	101,101,101,101	0
3	ZN	C	403	1/1	0.86	0.41	109,109,109,109	0
3	ZN	H	402	1/1	0.89	0.23	78,78,78,78	0
4	PO4	C	404	5/5	0.89	0.31	68,89,95,97	0
3	ZN	F	403	1/1	0.90	0.32	95,95,95,95	0
3	ZN	E	403	1/1	0.92	0.40	92,92,92,92	0
3	ZN	E	402	1/1	0.93	0.25	87,87,87,87	0
2	GOL	F	401	6/6	0.94	0.12	28,34,41,41	0
4	PO4	H	404	5/5	0.94	0.19	53,53,68,75	0
4	PO4	E	404	5/5	0.95	0.25	61,74,85,92	0
2	GOL	A	401	6/6	0.95	0.11	27,32,34,44	0
3	ZN	F	402	1/1	0.96	0.17	73,73,73,73	0
2	GOL	E	401	6/6	0.96	0.12	26,36,38,46	0
3	ZN	C	402	1/1	0.96	0.15	75,75,75,75	0

Continued on next page...

For Manuscript Review

Continued from previous page...

Mol	Type	Chain	Res	Atoms	RSCC	RSR	B-factors($Å^2$)	Q<0.9
2	GOL	B	401	6/6	0.96	0.11	29,33,37,38	0
3	ZN	D	402	1/1	0.96	0.24	76,76,76,76	0
3	ZN	A	402	1/1	0.96	0.17	76,76,76,76	0
4	PO4	D	404	5/5	0.96	0.20	53,56,67,68	0
2	GOL	C	401	6/6	0.96	0.14	28,33,36,40	0
4	PO4	F	404	5/5	0.96	0.18	57,65,69,69	0
3	ZN	B	402	1/1	0.96	0.23	83,83,83,83	0
2	GOL	D	401	6/6	0.97	0.15	27,33,34,43	0
4	PO4	A	404	5/5	0.97	0.18	43,45,61,66	0
4	PO4	B	404	5/5	0.97	0.20	57,66,81,83	0
4	PO4	G	404	5/5	0.97	0.20	56,66,73,78	0
2	GOL	H	401	6/6	0.97	0.10	28,30,33,37	0
3	ZN	G	402	1/1	0.98	0.17	76,76,76,76	0
2	GOL	G	401	6/6	0.98	0.10	27,28,31,39	0

6.5 Other polymers (i)

There are no such residues in this entry.

For Manuscript Review
