# Supplementary material for: Bioinformatics of cyanophycin metabolism genes and characterization of promiscuous isoaspartyl dipeptidases that catalyze the final step of cyanophycin degradation
Source: Sci Rep. 2023 May 23;13:8314. doi: 10.1038/s41598-023-34587-w (PMC10206079; doi:10.1038/s41598-023-34587-w)
Supplement: Supplementary file 2 — Supplementary Information 2. [file 41598_2023_34587_MOESM2_ESM.pdf]

Full wwPDB X-ray Structure Validation Report (i)

Aug 2, 2022 – 05:37 PM EDT

PDB ID	:	8DQM
Title	:	Crystal structure of isoaspartyl aminopeptidase from Roseivivax halodurans DSM 15395
Deposited on	:	2022-07-19
Resolution	:	2.70 Å(reported)

This wwPDB validation report is for manuscript review

This is a Full wwPDB X-ray Structure Validation Report.

This report is produced by the wwPDB biocuration pipeline after annotation of the structure.

We welcome your comments at *validation@mail.wwpdb.org*
A user guide is available at
https://www.wwpdb.org/validation/2017/XrayValidationReportHelp
with specific help available everywhere you see the (i) symbol.

The types of validation reports are described at http://www.wwpdb.org/validation/2017/FAQs#types.

The following versions of software and data (see references (i)) were used in the production of this report:

MolProbity	:	4.02b-467
Xtriage (Phenix)	:	1.13
EDS	:	2.29
Percentile statistics	:	20191225.v01 (using entries in the PDB archive December 25th 2019)
Refmac	:	5.8.0158
CCP4	:	7.0.044 (Gargrove)
Ideal geometry (proteins)	:	Engh & Huber (2001)
Ideal geometry (DNA, RNA)	:	Parkinson et al. (1996)
Validation Pipeline (wwPDB-VP)	:	2.29

For Manuscript Review

1 Overall quality at a glance (i)

The following experimental techniques were used to determine the structure:
X-RAY DIFFRACTION

The reported resolution of this entry is 2.70 Å.

Percentile scores (ranging between 0-100) for global validation metrics of the entry are shown in the following graphic. The table shows the number of entries on which the scores are based.

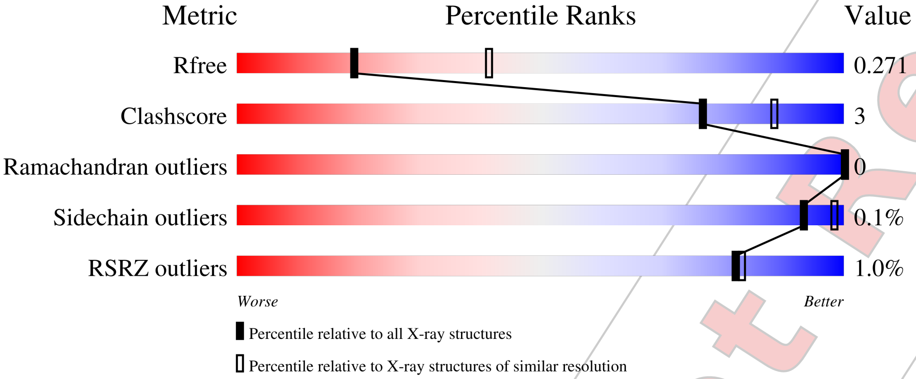

Metric	Whole archive (#Entries)	Similar resolution (#Entries, resolution range(Å))
R_{free}	130704	2808 (2.70-2.70)
Clashscore	141614	3122 (2.70-2.70)
Ramachandran outliers	138981	3069 (2.70-2.70)
Sidechain outliers	138945	3069 (2.70-2.70)
RSRZ outliers	127900	2737 (2.70-2.70)

The table below summarises the geometric issues observed across the polymeric chains and their fit to the electron density. The red, orange, yellow and green segments of the lower bar indicate the fraction of residues that contain outliers for >=3, 2, 1 and 0 types of geometric quality criteria respectively. A grey segment represents the fraction of residues that are not modelled. The numeric value for each fraction is indicated below the corresponding segment, with a dot representing fractions <=5% The upper red bar (where present) indicates the fraction of residues that have poor fit to the electron density. The numeric value is given above the bar.

Mol	Chain	Length	Quality of chain
1	A	177	% 84% 6% 10%
1	C	177	% 81% 7% 12%
1	E	177	2% 82% 7% 11%
1	G	177	% 88% • 8%
2	B	139	% 93% 5% •

Continued on next page...

For Manuscript Review

Continued from previous page...

Mol	Chain	Length	Quality of chain
2	D	139	% 88% 9% •
2	F	139	% 91% 5% •
2	H	139	96% •

For Manuscript Review

2 Entry composition (i)

There are 4 unique types of molecules in this entry. The entry contains 8662 atoms, of which 0 are hydrogens and 0 are deuteriums.

In the tables below, the ZeroOcc column contains the number of atoms modelled with zero occupancy, the AltConf column contains the number of residues with at least one atom in alternate conformation and the Trace column contains the number of residues modelled with at most 2 atoms.

- Molecule 1 is a protein called Isoaspartyl aminopeptidase.

Mol	Chain	Residues	Atoms					ZeroOcc	AltConf	Trace
1	A	159	Total 1169	C 717	N 219	O 226	S 7	0	0	0
1	C	156	Total 1147	C 705	N 215	O 220	S 7	0	0	0
1	E	157	Total 1151	C 707	N 216	O 221	S 7	0	0	0
1	G	162	Total 1188	C 728	N 222	O 231	S 7	0	0	0

- Molecule 2 is a protein called Isoaspartyl aminopeptidase.

Mol	Chain	Residues	Atoms					ZeroOcc	AltConf	Trace
2	B	136	Total 966	C 586	N 178	O 197	S 5	0	0	0
2	D	134	Total 950	C 576	N 175	O 194	S 5	0	0	0
2	F	133	Total 941	C 571	N 174	O 191	S 5	0	0	0
2	H	139	Total 998	C 609	N 182	O 202	S 5	0	0	0

There are 24 discrepancies between the modelled and reference sequences:

Chain	Residue	Modelled	Actual	Comment	Reference
B	313	GLU	-	expression tag	UNP X7EBZ8
B	314	ASN	-	expression tag	UNP X7EBZ8
B	315	LEU	-	expression tag	UNP X7EBZ8
B	316	TYR	-	expression tag	UNP X7EBZ8
B	317	PHE	-	expression tag	UNP X7EBZ8
B	318	GLN	-	expression tag	UNP X7EBZ8
D	313	GLU	-	expression tag	UNP X7EBZ8
D	314	ASN	-	expression tag	UNP X7EBZ8

Continued on next page...

For Manuscript Review

Continued from previous page...

Chain	Residue	Modelled	Actual	Comment	Reference
D	315	LEU	-	expression tag	UNP X7EBZ8
D	316	TYR	-	expression tag	UNP X7EBZ8
D	317	PHE	-	expression tag	UNP X7EBZ8
D	318	GLN	-	expression tag	UNP X7EBZ8
F	313	GLU	-	expression tag	UNP X7EBZ8
F	314	ASN	-	expression tag	UNP X7EBZ8
F	315	LEU	-	expression tag	UNP X7EBZ8
F	316	TYR	-	expression tag	UNP X7EBZ8
F	317	PHE	-	expression tag	UNP X7EBZ8
F	318	GLN	-	expression tag	UNP X7EBZ8
H	313	GLU	-	expression tag	UNP X7EBZ8
H	314	ASN	-	expression tag	UNP X7EBZ8
H	315	LEU	-	expression tag	UNP X7EBZ8
H	316	TYR	-	expression tag	UNP X7EBZ8
H	317	PHE	-	expression tag	UNP X7EBZ8
H	318	GLN	-	expression tag	UNP X7EBZ8

- Molecule 3 is SODIUM ION (three-letter code: NA) (formula: Na).

Mol	Chain	Residues	Atoms	ZeroOcc	AltConf
3	A	1	Total Na<br>1 1	0	0
3	C	1	Total Na<br>1 1	0	0
3	E	1	Total Na<br>1 1	0	0
3	G	1	Total Na<br>1 1	0	0

- Molecule 4 is water.

Mol	Chain	Residues	Atoms	ZeroOcc	AltConf
4	A	32	Total O<br>32 32	0	0
4	B	11	Total O<br>11 11	0	0
4	C	17	Total O<br>17 17	0	0
4	D	16	Total O<br>16 16	0	0
4	E	18	Total O<br>18 18	0	0

Continued on next page...

For Manuscript Review

Continued from previous page...

Mol	Chain	Residues	Atoms	ZeroOcc	AltConf
4	F	16	Total O<br>16 16	0	0
4	G	22	Total O<br>22 22	0	0
4	H	16	Total O<br>16 16	0	0

For Manuscript Review

3 Residue-property plots (i)

These plots are drawn for all protein, RNA, DNA and oligosaccharide chains in the entry. The first graphic for a chain summarises the proportions of the various outlier classes displayed in the second graphic. The second graphic shows the sequence view annotated by issues in geometry and electron density. Residues are color-coded according to the number of geometric quality criteria for which they contain at least one outlier: green = 0, yellow = 1, orange = 2 and red = 3 or more. A red dot above a residue indicates a poor fit to the electron density (RSRZ > 2). Stretches of 2 or more consecutive residues without any outlier are shown as a green connector. Residues present in the sample, but not in the model, are shown in grey.

- Molecule 1: Isoaspartyl aminopeptidase

Chain A:

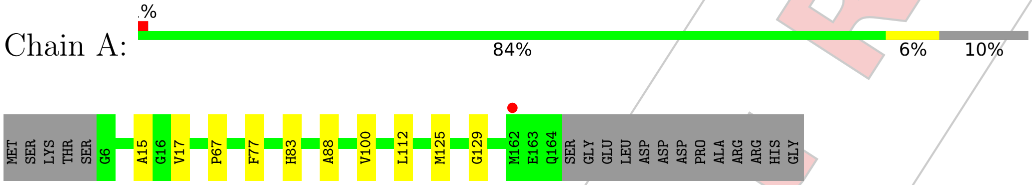

- Molecule 1: Isoaspartyl aminopeptidase

Chain C:

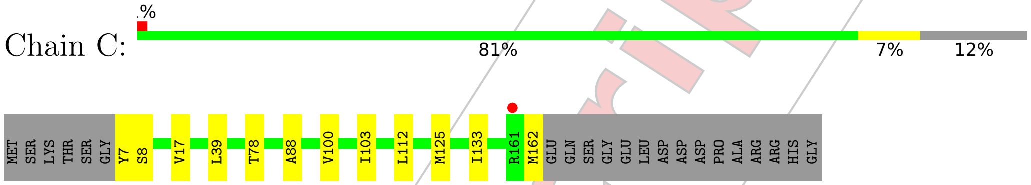

- Molecule 1: Isoaspartyl aminopeptidase

Chain E:

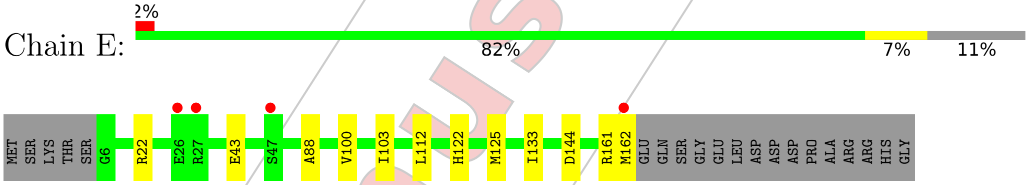

- Molecule 1: Isoaspartyl aminopeptidase

Chain G:

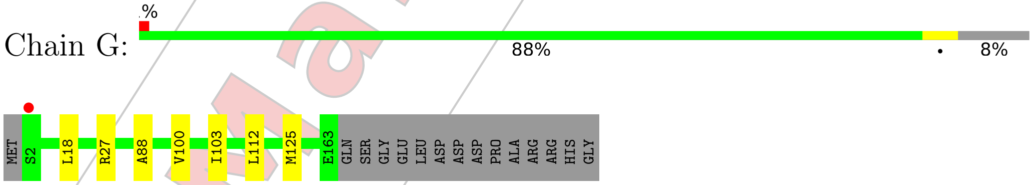

- Molecule 2: Isoaspartyl aminopeptidase

Chain B:

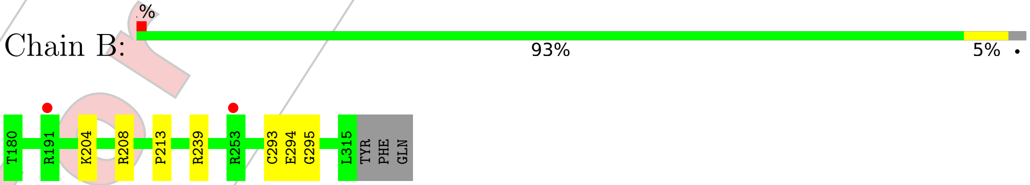

- Molecule 2: Isoaspartyl aminopeptidase

For Manuscript Review

Chain D: 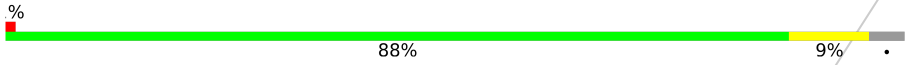

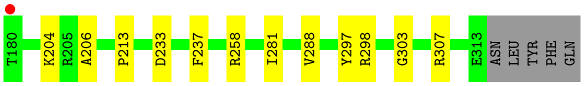

• Molecule 2: Isoaspartyl aminopeptidase

Chain F: 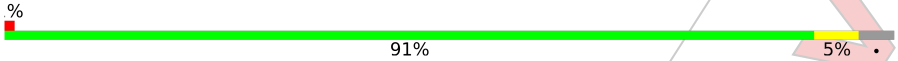

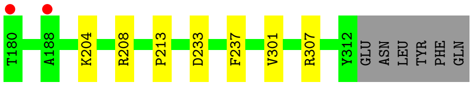

• Molecule 2: Isoaspartyl aminopeptidase

Chain H: 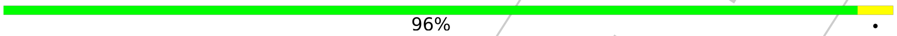

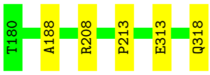

For Manuscript Review

4 Data and refinement statistics (i)

Property	Value	Source
Space group	P 21 21 21	Depositor
Cell constants<br>a, b, c, α, β, γ	62.22Å 154.59Å 197.91Å<br>90.00° 90.00° 90.00°	Depositor
Resolution (Å)	98.96 – 2.70<br>121.83 – 2.70	Depositor<br>EDS
% Data completeness<br>(in resolution range)	99.9 (98.96-2.70)<br>100.0 (121.83-2.70)	Depositor<br>EDS
R_{merge}	0.04	Depositor
R_{sym}	(Not available)	Depositor
$< I/\sigma(I) >$ [1]	1.48 (at 2.69Å)	Xtriage
Refinement program	PHENIX (1.20.1_4487)	Depositor
R, R_{free}	0.243 , 0.268<br>0.245 , 0.271	Depositor<br>DCC
R_{free} test set	2637 reflections (4.94%)	wwPDB-VP
Wilson B-factor (Å^2)	39.5	Xtriage
Anisotropy	0.783	Xtriage
Bulk solvent k_{sol}(e/Å^3), B_{sol}(Å^2)	0.34 , 33.4	EDS
L-test for twinning[2]	$< \|L\| > = 0.43$, $< L^2 > = 0.25$	Xtriage
Estimated twinning fraction	No twinning to report.	Xtriage
F_o,F_c correlation	0.90	EDS
Total number of atoms	8662	wwPDB-VP
Average B, all atoms (Å^2)	41.0	wwPDB-VP

Xtriage's analysis on translational NCS is as follows: *The analyses of the Patterson function reveals a significant off-origin peak that is 20.60 % of the origin peak, indicating pseudo-translational symmetry. The chance of finding a peak of this or larger height randomly in a structure without pseudo-translational symmetry is equal to 8.3866e-03. The detected translational NCS is most likely also responsible for the elevated intensity ratio.*

[1] Intensities estimated from amplitudes.

[2] Theoretical values of $< |L| >$, $< L^2 >$ for acentric reflections are 0.5, 0.333 respectively for untwinned datasets, and 0.375, 0.2 for perfectly twinned datasets.

For Manuscript Review

5 Model quality (i)

5.1 Standard geometry (i)

Bond lengths and bond angles in the following residue types are not validated in this section: NA

The Z score for a bond length (or angle) is the number of standard deviations the observed value is removed from the expected value. A bond length (or angle) with $|Z| > 5$ is considered an outlier worth inspection. RMSZ is the root-mean-square of all Z scores of the bond lengths (or angles).

Mol	Chain	Bond lengths		Bond angles	
		RMSZ	#$\|Z\|$ >5	RMSZ	#$\|Z\|$ >5
1	A	0.67	0/1185	0.79	0/1598
1	C	0.66	0/1163	0.77	0/1569
1	E	0.67	0/1167	0.76	0/1574
1	G	0.65	0/1204	0.76	0/1623
2	B	0.67	0/975	0.85	0/1315
2	D	0.67	0/959	0.85	0/1293
2	F	0.67	0/950	0.83	0/1281
2	H	0.66	0/1009	0.84	0/1361
All	All	0.66	0/8612	0.80	0/11614

There are no bond length outliers.

There are no bond angle outliers.

There are no chirality outliers.

There are no planarity outliers.

5.2 Too-close contacts (i)

In the following table, the Non-H and H(model) columns list the number of non-hydrogen atoms and hydrogen atoms in the chain respectively. The H(added) column lists the number of hydrogen atoms added and optimized by MolProbity. The Clashes column lists the number of clashes within the asymmetric unit, whereas Symm-Clashes lists symmetry-related clashes.

Mol	Chain	Non-H	H(model)	H(added)	Clashes	Symm-Clashes
1	A	1169	0	1141	8	0
1	C	1147	0	1124	11	0
1	E	1151	0	1127	12	0
1	G	1188	0	1163	5	0
2	B	966	0	943	6	0

Continued on next page...

For Manuscript Review

Continued from previous page...

Mol	Chain	Non-H	H(model)	H(added)	Clashes	Symm-Clashes
2	D	950	0	926	12	0
2	F	941	0	920	5	0
2	H	998	0	969	6	0
3	A	1	0	0	0	0
3	C	1	0	0	0	0
3	E	1	0	0	0	0
3	G	1	0	0	0	0
4	A	32	0	0	1	0
4	B	11	0	0	0	0
4	C	17	0	0	1	0
4	D	16	0	0	1	0
4	E	18	0	0	2	0
4	F	16	0	0	0	0
4	G	22	0	0	0	0
4	H	16	0	0	4	0
All	All	8662	0	8313	51	0

The all-atom clashscore is defined as the number of clashes found per 1000 atoms (including hydrogen atoms). The all-atom clashscore for this structure is 3.

All (51) close contacts within the same asymmetric unit are listed below, sorted by their clash magnitude.

Atom-1	Atom-2	Interatomic distance (Å)	Clash overlap (Å)
1:E:22:ARG:NH1	4:E:303:HOH:O	2.18	0.68
2:D:233:ASP:O	2:D:237:PHE:HD1	1.78	0.67
2:H:188:ALA:CB	4:H:405:HOH:O	2.44	0.66
1:E:144:ASP:O	4:E:302:HOH:O	2.15	0.64
2:B:204:LYS:HD2	2:B:208:ARG:HG3	1.81	0.63
1:A:67:PRO:O	4:A:301:HOH:O	2.17	0.60
1:A:100:VAL:HG11	1:A:112:LEU:HG	1.84	0.58
1:G:100:VAL:HG11	1:G:112:LEU:HG	1.85	0.58
1:E:100:VAL:HG11	1:E:112:LEU:HG	1.87	0.56
1:G:103:ILE:HD13	1:G:112:LEU:HD23	1.89	0.54
1:C:100:VAL:HG11	1:C:112:LEU:HG	1.89	0.53
2:H:188:ALA:HB2	4:H:405:HOH:O	2.06	0.53
2:H:313:GLU:HA	4:H:410:HOH:O	2.09	0.52
2:H:318:GLN:NE2	4:H:402:HOH:O	2.22	0.52
1:C:17:VAL:HA	2:D:297:TYR:CZ	2.45	0.52
2:B:239:ARG:CD	4:C:302:HOH:O	2.58	0.51
1:E:43:GLU:CD	2:F:301:VAL:HG21	2.30	0.51
1:E:122:HIS:CD2	2:H:208:ARG:HG3	2.45	0.50

Continued on next page...

For Manuscript Review

Continued from previous page...

Atom-1	Atom-2	Interatomic distance (Å)	Clash overlap (Å)
1:C:88:ALA:HB2	2:D:213:PRO:HA	1.93	0.50
1:E:161:ARG:O	1:E:162:MET:HB2	2.10	0.50
1:E:161:ARG:O	1:E:162:MET:CB	2.60	0.49
2:D:298:ARG:NE	4:D:402:HOH:O	2.13	0.49
1:C:78:THR:HG22	2:D:204:LYS:HE2	1.94	0.49
1:C:103:ILE:HD13	1:C:112:LEU:HD23	1.94	0.49
1:E:103:ILE:HD13	1:E:112:LEU:HD23	1.93	0.48
1:A:17:VAL:HG21	2:B:295:GLY:HA3	1.95	0.47
1:C:125:MET:SD	1:C:133:ILE:CD1	3.02	0.47
1:A:15:ALA:HB1	2:B:293:CYS:SG	2.54	0.47
2:D:258:ARG:HG2	2:D:288:VAL:CG1	2.45	0.47
1:E:125:MET:SD	1:E:133:ILE:HD11	2.54	0.47
1:E:125:MET:SD	1:E:133:ILE:CD1	3.03	0.47
2:B:294:GLU:OE1	2:B:294:GLU:N	2.37	0.47
2:F:233:ASP:O	2:F:237:PHE:HD1	2.00	0.44
1:A:88:ALA:HB2	2:B:213:PRO:HA	2.00	0.43
2:D:258:ARG:HG2	2:D:288:VAL:HG12	2.00	0.43
2:D:233:ASP:O	2:D:237:PHE:CD1	2.65	0.43
1:C:125:MET:SD	1:C:133:ILE:HD11	2.58	0.43
1:G:18:LEU:HD22	1:G:27:ARG:HD3	1.99	0.43
2:F:204:LYS:HD2	2:F:208:ARG:HD2	2.01	0.43
1:A:129:GLY:HA3	2:D:206:ALA:O	2.18	0.43
1:A:77:PHE:CE1	1:A:83:HIS:CE1	3.07	0.43
1:E:88:ALA:HB2	2:F:213:PRO:HA	2.00	0.42
1:C:8:SER:HB2	2:D:281:ILE:HD11	2.01	0.42
1:G:88:ALA:HB2	2:H:213:PRO:HA	2.02	0.42
1:A:100:VAL:HA	1:A:125:MET:O	2.20	0.42
1:G:100:VAL:HA	1:G:125:MET:O	2.20	0.41
1:C:100:VAL:HA	1:C:125:MET:O	2.20	0.41
1:E:100:VAL:HA	1:E:125:MET:O	2.21	0.41
1:C:7:TYR:CZ	2:D:303:GLY:HA2	2.56	0.41
1:C:39:LEU:HD13	2:D:307:ARG:HG3	2.03	0.41
2:F:301:VAL:HG23	2:F:307:ARG:CG	2.51	0.40

There are no symmetry-related clashes.

For Manuscript Review

5.3 Torsion angles (i)

5.3.1 Protein backbone (i)

In the following table, the Percentiles column shows the percent Ramachandran outliers of the chain as a percentile score with respect to all X-ray entries followed by that with respect to entries of similar resolution.

The Analysed column shows the number of residues for which the backbone conformation was analysed, and the total number of residues.

Mol	Chain	Analysed	Favoured	Allowed	Outliers	Percentiles	
1	A	157/177 (89%)	154 (98%)	3 (2%)	0	100	100
1	C	154/177 (87%)	150 (97%)	4 (3%)	0	100	100
1	E	155/177 (88%)	151 (97%)	4 (3%)	0	100	100
1	G	160/177 (90%)	157 (98%)	3 (2%)	0	100	100
2	B	134/139 (96%)	128 (96%)	6 (4%)	0	100	100
2	D	132/139 (95%)	125 (95%)	7 (5%)	0	100	100
2	F	131/139 (94%)	125 (95%)	6 (5%)	0	100	100
2	H	137/139 (99%)	128 (93%)	9 (7%)	0	100	100
All	All	1160/1264 (92%)	1118 (96%)	42 (4%)	0	100	100

There are no Ramachandran outliers to report.

5.3.2 Protein sidechains (i)

In the following table, the Percentiles column shows the percent sidechain outliers of the chain as a percentile score with respect to all X-ray entries followed by that with respect to entries of similar resolution.

The Analysed column shows the number of residues for which the sidechain conformation was analysed, and the total number of residues.

Mol	Chain	Analysed	Rotameric	Outliers	Percentiles	
1	A	111/126 (88%)	111 (100%)	0	100	100
1	C	109/126 (86%)	108 (99%)	1 (1%)	78	92
1	E	109/126 (86%)	109 (100%)	0	100	100
1	G	114/126 (90%)	114 (100%)	0	100	100
2	B	92/95 (97%)	92 (100%)	0	100	100
2	D	90/95 (95%)	90 (100%)	0	100	100

Continued on next page...

For Manuscript Review

Continued from previous page...

Mol	Chain	Analysed	Rotameric	Outliers	Percentiles	
2	F	89/95 (94%)	89 (100%)	0	100	100
2	H	95/95 (100%)	95 (100%)	0	100	100
All	All	809/884 (92%)	808 (100%)	1 (0%)	93	98

All (1) residues with a non-rotameric sidechain are listed below:

Mol	Chain	Res	Type
1	C	162	MET

Sometimes sidechains can be flipped to improve hydrogen bonding and reduce clashes. All (2) such sidechains are listed below:

Mol	Chain	Res	Type
1	A	83	HIS
2	D	244	HIS

5.3.3 RNA (i)

There are no RNA molecules in this entry.

5.4 Non-standard residues in protein, DNA, RNA chains (i)

There are no non-standard protein/DNA/RNA residues in this entry.

5.5 Carbohydrates (i)

There are no monosaccharides in this entry.

5.6 Ligand geometry (i)

Of 4 ligands modelled in this entry, 4 are monoatomic - leaving 0 for Mogul analysis.

There are no bond length outliers.

There are no bond angle outliers.

There are no chirality outliers.

There are no torsion outliers.

There are no ring outliers.

For Manuscript Review

No monomer is involved in short contacts.

5.7 Other polymers (i)

There are no such residues in this entry.

5.8 Polymer linkage issues (i)

There are no chain breaks in this entry.

For Manuscript Review

6 Fit of model and data (i)

6.1 Protein, DNA and RNA chains (i)

In the following table, the column labelled '#RSRZ> 2' contains the number (and percentage) of RSRZ outliers, followed by percent RSRZ outliers for the chain as percentile scores relative to all X-ray entries and entries of similar resolution. The OWAB column contains the minimum, median, 95th percentile and maximum values of the occupancy-weighted average B-factor per residue. The column labelled 'Q< 0.9' lists the number of (and percentage) of residues with an average occupancy less than 0.9.

Mol	Chain	Analysed	<RSRZ>	#RSRZ>2			OWAB(Å^2)	Q<0.9
1	A	159/177 (89%)	0.50	1 (0%)	89	91	26, 34, 53, 79	0
1	C	156/177 (88%)	0.53	1 (0%)	89	91	32, 40, 61, 84	0
1	E	157/177 (88%)	0.55	4 (2%)	57	59	29, 42, 65, 92	0
1	G	162/177 (91%)	0.48	1 (0%)	89	91	24, 34, 56, 76	0
2	B	136/139 (97%)	0.49	2 (1%)	73	76	23, 35, 50, 58	0
2	D	134/139 (96%)	0.66	1 (0%)	87	89	27, 43, 57, 66	0
2	F	133/139 (95%)	0.54	2 (1%)	73	76	30, 45, 58, 68	0
2	H	139/139 (100%)	0.44	0	100	100	25, 35, 50, 63	0
All	All	1176/1264 (93%)	0.52	12 (1%)	82	83	23, 39, 58, 92	0

All (12) RSRZ outliers are listed below:

Mol	Chain	Res	Type	RSRZ
1	G	2	SER	4.1
1	E	27	ARG	3.6
2	D	180	THR	3.5
1	E	47	SER	2.8
2	F	180	THR	2.3
1	E	26	GLU	2.2
1	A	162	MET	2.2
1	E	162	MET	2.2
2	B	253	ARG	2.1
2	F	188	ALA	2.0
1	C	161	ARG	2.0
2	B	191	ARG	2.0

For Manuscript Review

6.2 Non-standard residues in protein, DNA, RNA chains (i)

There are no non-standard protein/DNA/RNA residues in this entry.

6.3 Carbohydrates (i)

There are no monosaccharides in this entry.

6.4 Ligands (i)

In the following table, the Atoms column lists the number of modelled atoms in the group and the number defined in the chemical component dictionary. The B-factors column lists the minimum, median, 95th percentile and maximum values of B factors of atoms in the group. The column labelled 'Q< 0.9' lists the number of atoms with occupancy less than 0.9.

Mol	Type	Chain	Res	Atoms	RSCC	RSR	B-factors($Å^2$)	Q<0.9
3	NA	C	201	1/1	0.89	0.10	38,38,38,38	0
3	NA	A	201	1/1	0.92	0.26	71,71,71,71	0
3	NA	E	201	1/1	0.97	0.14	41,41,41,41	0
3	NA	G	201	1/1	0.99	0.12	29,29,29,29	0

6.5 Other polymers (i)

There are no such residues in this entry.

For Manuscript Review
